# Some Human Lessons in War-Time

**Published:** 1916-09-23

**Authors:** 


### LIFE AND DEATH.

### Some Human Lessons in War Time.

### I. THE NATION AND THE GREAT WAR.

During the last few months we have been publishing a series of studies in human temperament and these have attracted a great deal of attention. In them the mental attitudes of various types of men and women have been critically examined, and individual idiosyncrasies and vagaries have been reviewed. Such studies, so far from being alien to the spirit of this journal, are an essential part of our programme. "Know then thyself . . . the proper study of mankind is man," sang Pope, and in this respect we cordially agree with him. Indeed, one great purpose which The Hospital was founded to fulfil was to give every intelligent member of the race, man or woman, the means whereby self-knowledge could be obtained, and this with the object of enabling each to live in the soundest possible health, and thus to avoid the degeneracy of adopting a lower standard and a fall to the level of mere existence.

The war came upon the nation like an avalanche: from the Government downwards no one was prepared for it, and consequently everybody was taken at a great disadvantage. It is not our purpose in this series of articles to examine causes, but rather to state facts. For several decades the people in Great Britain had been increasingly prosperous; money was abundant; and wealth, in the sense of the possession of more than enough to each day's needs, was general. Evidence would have been still more plain had it not been for the loss in many classes of the people of the spirit of independence and self-respect, such loss resulting from a desire to outshine their neighbours in outward display, with consequent expenditure beyond their means. The war has steadily revealed how widespread was the degeneracy arising from the cause just mentioned, and the whole extent of the mischief is not even yet revealed, though the war is making it plainer and plainer every week.

So deep-seated was the self-satisfaction of the majority of the people in these islands that when war was declared not a third of the population appreciated the significance of the fact, or were prepared to take the situation seriously and to devote themselves to the defence of their country and the upholding of its reputation and independence against all comers. Slowly, but surely, the people began to realise the terrible character of the war; and the true inwardness of those who commenced it, with the purpose of reducing the populations of many countries to a condition approaching slavery, became evident. All classes then grasped that this slowly matured and carefully organised policy was to be enforced by the use of the immense army and war material which Germany had built up during forty years of continuous effort, and that the military leaders had set themselves deliberately to organise a machine to destroy the freedom of other nations and to reduce these to a state of subjection to the German Emperor.

The continuous absence of a great war, which adversely affected the life and interests of the British people, and the existence of a belief that Britain would always muddle through whatever the difficulties might be, made it at first impossible, and, even later, extremely difficult, for the people of these islands to appreciate the fact that they were engaged in the greatest war the world has yet seen. Further, that not only the British nation, but also the British Empire was in grave peril, a peril which could only be resisted and overcome by the devoted efforts of every patriotic man and every earnest woman in these islands. After two years the people as a whole woke up to the true position, and to-day the unity of our citizens, the splendid fibre and cheerfulness of our sailors and soldiers, and the self-denying co-operation of all classes of men and women with our fighting men have placed our warriors and Allies in a position strong enough to repel the foe, and to render him henceforward impotent for mischief to other nations. This war has afforded magnificent proof of the sterling value of nationality and race, and of the glorious fact that our people are worthy descendants of their forbears and sires—of the men who made England a nation and laid the foundation of an Empire on which the sun never sets.

It is true, therefore, to affirm that Britons are to-day fully awake and increasingly alive to the existing situation. Unfortunately there are still a mass of people who have not yet taken any part directly or indirectly in the war, several of whom are neither an ornament nor a credit to the nation or to themselves. It takes many kinds of people to make a world, but England would be all the better if the small group of so-called pacifists, some of them members of Parliament, were to be put in a reservation camp and fed upon the bread and water of affliction until the war is over. The same course might usefully be followed in the case of spurious conscientious objectors, who have been demonstrated as mere shirkers, and so too degenerate for the British Army and unworthy to enjoy longer the protection of the British race. England is known all the world over as a land of refuge for every cast-out member of every race who may flee to it. This spirit of universal hospitality has been gravely abused from time to time, and the gravest examples of this abuse have been manifest, since the war began, in the presence and protection of persons who have systematically plotted against the safety and welfare of the British people.

Not a few of such persons, there is good reason to believe, have probably received, and may still be receiving, gifts of money from the enemies of the nation.

We have given this terse summary of the position of the nation alike at the outset of the war, during its continuation, and at the present time, as a necessary introduction to a series of articles dealing with human lessons in war-time, and we hope these articles may prove not only interesting but also helpful in the direction of indicating the social revolution which may result through the great war. The organic changes which are likely to follow on the declaration of peace should have the serious attention of everybody interested in the nation and Empire. Our purpose will be to drive home the importance, for each individual, of cultivating life, as opposed to mere existence; and of health, as a paramount factor in the maintenance of a maximum vitality. We shall examine also the factors in daily life which respectively help and hinder these much to be desired ends.

